# Ilimaquinone (Marine Sponge Metabolite) Induces Apoptosis in HCT-116 Human Colorectal Carcinoma Cells via Mitochondrial-Mediated Apoptosis Pathway

**DOI:** 10.3390/md20090582

**Published:** 2022-09-18

**Authors:** Malvi Surti, Mitesh Patel, Alya Redhwan, Lamya Ahmed Al-Keridis, Mohd Adnan, Nawaf Alshammari, Mandadi Narsimha Reddy

**Affiliations:** 1 Bapalal Vaidya Botanical Research Centre, Department of Biosciences, Veer Narmad South Gujarat University, Surat 395007, India; 2 Department of Biotechnology, Parul Institute of Applied Sciences and Centre of Research for Development, Parul University, Vadodara 391760, India; 3 Department of Health, College of Health and Rehabilitation Sciences, Princess Nourah bint Abdulrahman University, P.O. Box 84428, Riyadh 11671, Saudi Arabia; 4 Department of Biology, College of Science, Princess Nourah bint Abdulrahman University, P.O. Box 84428, Riyadh 11671, Saudi Arabia; 5 Department of Biology, College of Science, University of Hail, Hail P.O. Box 2440, Saudi Arabia

**Keywords:** Ilimaquinone, colon cancer, apoptosis, marine sponge, HCT-116 cancer cell line, mitochondrial-mediated apoptosis pathway

## Abstract

Ilimaquinone (IQ), a metabolite found in marine sponges, has been reported to have a number of biological properties, including potential anticancer activity against colon cancer. However, no clear understanding of the precise mechanism involved is known. The aim of this study was to examine the molecular mechanism by which IQ acts on HCT-116 cells. The anticancer activity of IQ was investigated by means of a cell viability assay followed by the determination of induction of apoptosis by means of the use of acridine orange–ethidium bromide (AO/EB) staining, Annexin V/PI double staining, DNA fragmentation assays, and TUNEL assays. The mitochondrial membrane potential (ΔΨm) was detected using the JC-1 staining technique, and the apoptosis-associated proteins were analyzed using real-time qRT-PCR. A molecular docking study of IQ with apoptosis-associated proteins was also conducted in order to assess the interaction between IQ and them. Our results suggest that IQ significantly suppressed the viability of HCT-116 cells in a dose-dependent manner. Fluorescent microscopy, flow cytometry, DNA fragmentation and the TUNEL assay in treated cells demonstrated apoptotic death mode. As an additional confirmation of apoptosis, the increased level of caspase-3 and caspase-9 expression and the downregulation of Bcl-2 and mitochondrial dysfunction were observed in HCT-116 cells after treatment with IQ, which was accompanied by a decrease in mitochondrial membrane potential (ΔΨm). Overall, the results of our studies demonstrate that IQ could trigger mitochondria-mediated apoptosis as demonstrated by a decrease in ΔΨm, activation of caspase-9/-3, damage of DNA and a decrease in the proportion of Bcl-2 through the mitochondrial-mediated apoptosis pathway.

## 1. Introduction

At the present time, the occurrence of colorectal cancer (CRC) has increased dramatically, and it has become the third most common cancer type. Due to the unmet screening and therapeutic strategies, along with the increasing incidence rates, this disease also became the world’s second leading cause of cancer death. In 2020, CRC accounted for 10% of the global cancer incidence and 9.4% of cancer deaths [1]. On the basis of population growth, aging and human development projections, it is expected that there will be around 3.2 million new cases of CRC worldwide by 2040. The increasing incidence of CRC is primarily explained by increased exposure to environmental risk factors caused by changing lifestyles and diets toward Westernization [2].

Conventional treatments to treat cancer are still the most common option of treatment, despite the fact that they are mostly unsuccessful and lead to many deaths due to their side effects. Nevertheless, research into developing new drugs from natural sources that have fewer side effects is a very promising area for cancer research [3]. In the past few decades, natural compounds derived from sponges have increasingly attracted attention as potential innovative tools against cancer, since they are the sources of the majority of bioactive compounds [4]. As a consequence of the structurally varied varieties of metabolites obtained from sponges, they exhibit significant therapeutic potential for treating human diseases, making them well-suited for the studies of marine natural product chemists [5]. In contrast, bioactive compounds derived from marine organisms have a typically complex chemical structure, and their effects on the body affect multiple organs, functions, and targets; therefore, studying their mechanisms of action is very challenging [6]. 

Ilimaquinone (IQ) is one of the sesquiterpene quinines that was firstly isolated from the *Hippospongia metachromia* de Laubenfels marine sponge [7]. Quinone derivatives are well-known for their cytotoxicity via various mechanisms, such as redox cycling, arylation, intercalation, DNA strand breaks, free radical generation and alkylation. Anthracyclines (doxorubicin and mitoxanthrone) are the best-known examples of quinones with antitumor activity and inhibitory activity against type II topoisomerase. There has been a steady increase in studies devoted to the biological activity of natural and semisynthetic quinones [8]. According to the reports, it exhibits a variety of biological properties, including anti-HIV, antimicrobial, antimalarial, anti-inflammatory, antimitotic, antisecretory and anticancer properties [9,10,11,12,13,14]. Additionally, it has been documented that it is capable of causing Golgi fragmentation. This combination of biological properties makes it a promising candidate for further study. The cytotoxicity of IQ has been reported against few types of cancer cell lines [15,16,17]. In spite of this, further investigation is needed to establish the mechanism by which IQ induces cell death. 

In order to maintain the growth and homeostasis of tissues, apoptosis, or programmed cell death, plays an important role in regulating the number of cells in tissues in order to remove old, damaged and unwanted cells to maintain the growth and homeostasis of those tissues. In spite of the fact that this process is self-destructive, it is vital to the development and remodeling of tissues, immunoregulatory processes and several diseases [18]. Cells of eukaryotes have to balance survival signals and death signals that originate from their extracellular domains in order to maintain homeostasis. The process of cancer development is characterized by the non-initiation of apoptosis by the dividing tumor cells after DNA damage, and this is one of the primary characteristics of cancer development [19]. Apoptosis-inducing approaches could be useful in the treatment of cancer by reactivating the apoptotic machinery within tumor cells. The primary mechanism of action of most anticancer medications is to induce apoptosis in neoplastic cells. A number of natural ingredients have also been shown to possess promising anticancer properties or cancer-preventing effects by activating apoptosis pathways in carcinogenic cells [20]. This study aimed to assess the anticancer properties of IQ in colorectal cancer cells as well as its molecular mechanism for causing apoptosis. 

## 2. Results

### 2.1. Induction of Cytotoxicity by IQ in HCT-116 Cells 

**Figure 1:**
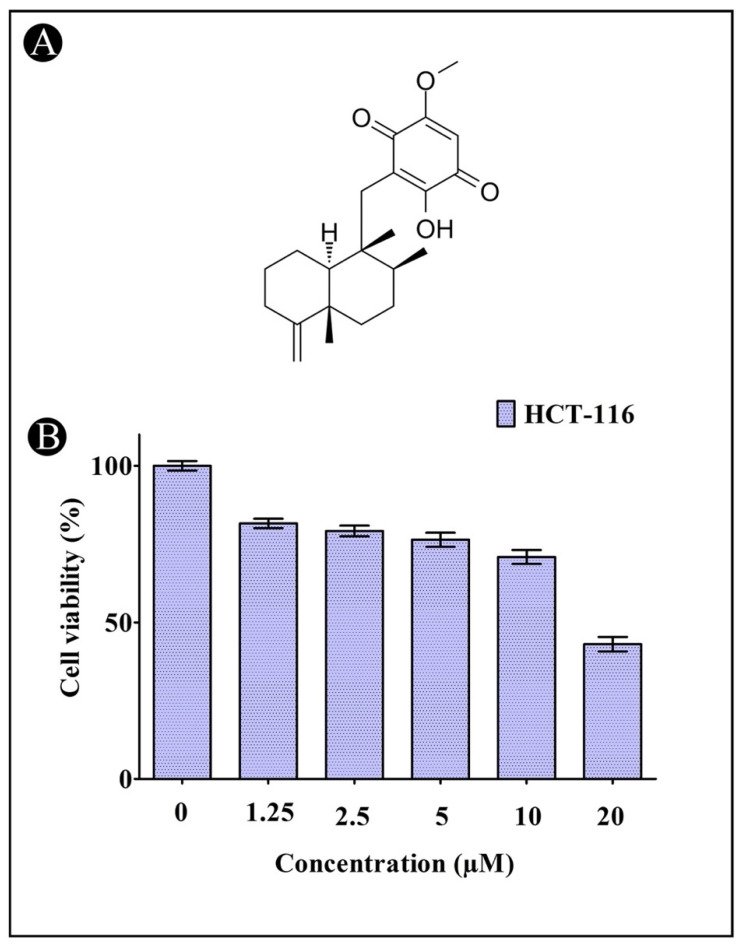
(**A**) The chemical structure of Ilimaquinone. (**B**) Cytotoxic activity of IQ against HCT-116 cells. Error bars indicate the SD (standard deviation) of three independent experiments.

Cell viability and proliferation were assessed using the MTT assay. In this assay, yellow tetrazolium MTT is reduced by mitochondrial succinate dehydrogenase into a purple formazan. The growth inhibitory effect of IQ was observed in HCT-116 cancer cells with an IC_50_ of 17.89 µM, which indicates that IQ inhibits cell proliferation in a dose-dependent manner (Figure 1A,B).

**Figure 2:**
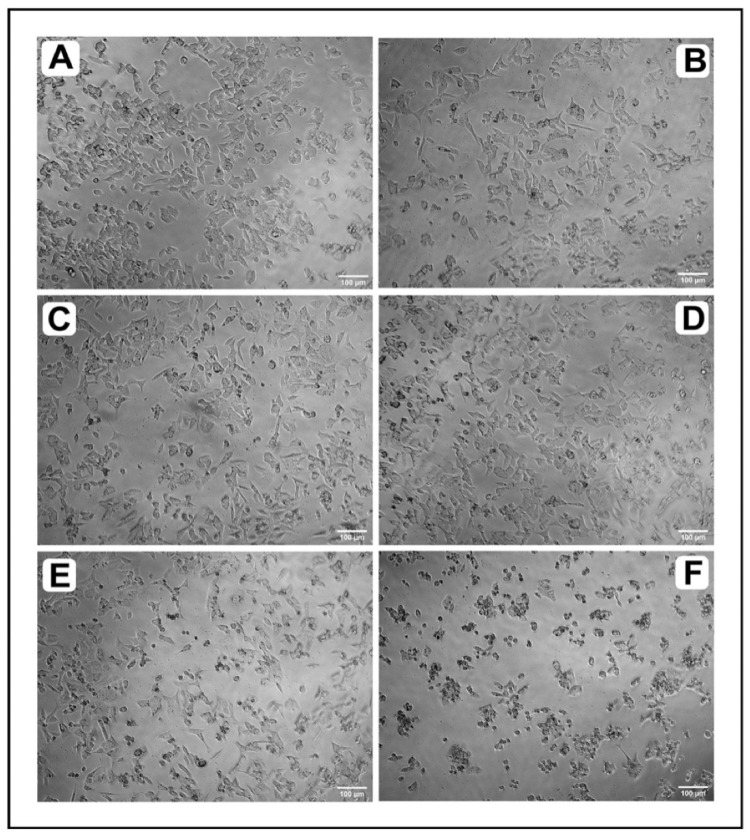
Morphological analysis of HCT-116 cells under inverted microscope after treatment with different concentrations of IQ with morphological changes. (**A**) Untreated, (**B**) 1.25 µM, (**C**) 2.5 µM, (**D**) 5 µM, (**E**) 10 µM and (**F**) 20 µM.

Likewise, to confirm that IQ is capable of inhibiting growth, we tested its effect of on the confluency of HCT-116 cells using the inverted microscope method. As depicted in Figure 2A–F, treatment with increased concentrations of IQ caused a gradual decrease in the confluency of the monolayer of HCT-116 cells. Cells also rounded, shrank and lost their alignment, and the intercellular spaces became larger.

### 2.2. Apoptosis Induction by IQ

#### 2.2.1. Microscopic Analysis

**Figure 3:**
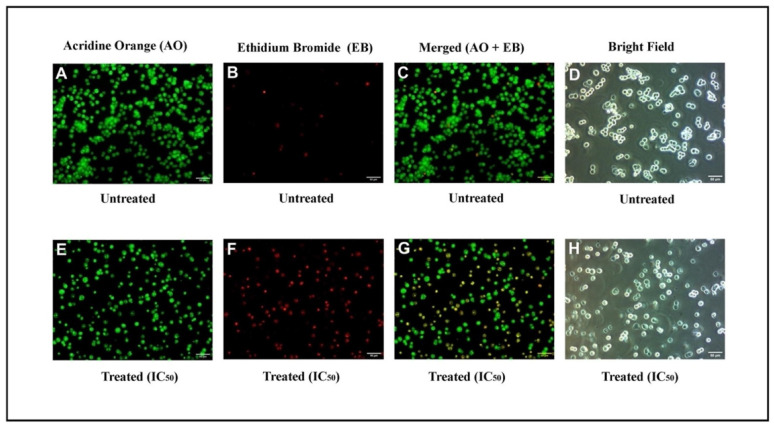
Fluorescence and bright-field microscopic studies of HCT-116 cells. (**A**–**D**) Untreated cells. (**E**–**H**) Cells exposed to IC_50_ of IQ for 24 h. For fluorescence microscopy, cells were stained using AO and EB.

In order to determine if apoptosis is caused by IQ, fluorescent microscopy analyses were performed using AO/EB-stained cells. Using the staining method with the cells, it was evident that the morphology of cancer cells treated with this method showed clear signs of apoptosis. The green cells that were stained with only AO represented viable cells, whereas the orange cells stained with EB were late apoptotic cells. On the other hand, the cells that were green and orange, with condensed chromatin, and those which were stained with AO and EB, were cells that have gone through an early apoptosis. As it was shown in the results of experiments carried out with HCT-116 cells treated with IQ, apoptosis is the most prevalent, being characterized by chromatin condensation as well as the degradation of the cell membrane (Figure 3A–H).

#### 2.2.2. Annexin V–Propidium Iodide (PI) Staining

**Figure 4:**
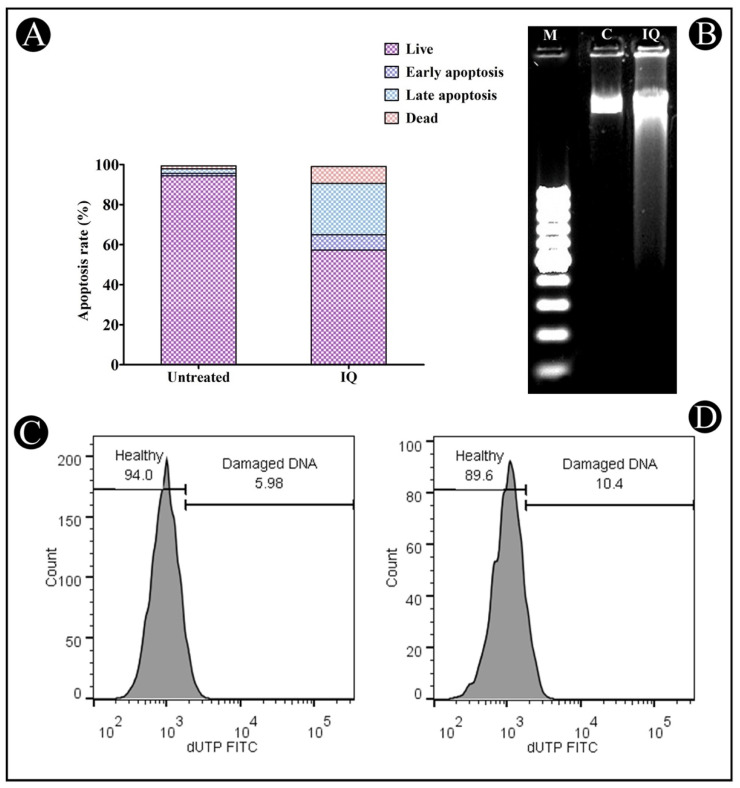
(**A**) Distribution of HCT-116 cells without treatment and treatment with IC_50_ concentration of IQ determined by Annexin V–PI apoptosis assay. (**B**) DNA fragmentation in treated HCT-116, Lane 1 (M): molecular marker, Lane 2 (C): control, Lane 3 (IQ): the cells treated with IQ (IC_50_), (**C**,**D**) DNA fragmentation in treated HCT-116 cells. Apoptotic intensity of HCT-116 cells was determined by flow cytometry after TUNEL assay. Shift of the population to the right in treated cells compared to control cells indicates the apoptotic cell population.

The amount of phosphatidylserine (PS) occurring within a healthy cell is restricted to the inner leaflet of the plasma membrane and is exposed to the cytoplasm. During the process of apoptosis, the outer leaflet of membrane of the cell is ruptured, leaving PS exposed on its outer leaflet as a result of the rupture in the membrane. By fluorescently labeling Annexin V (AnnexinV-FITC), which is a calcium-binding protein of 36 kDa, it is able to detect PS in apoptotic cells. Nevertheless, Annexin V is also capable of accessing the necrotic cell membrane, owing to the fact that these membranes have lost their integrity and are likely to allow Annexin V to pass through damaged plasma membranes. At the late stage of apoptosis, PI stains necrotic or apoptotic cells. In this way, viable, early and late apoptotic and necrotic cells can be distinguished by co-staining Annexin V-FITC and PI. In consequence, flow cytometry is capable of measuring the degree of apoptosis in treated cells by means of Annexin V binding and PI uptake and can distinguish between early apoptotic and late apoptotic/necrotic cells in the treated cells. Using Annexin V–PI staining, it was determined that IQ induces 7.62% early apoptosis, 25.74% late apoptosis and 8.37% of cell death in HCT-116 cells after 24 h of incubation (Figure 4A). Based on these results, it is clear that IQ is able to induce apoptosis in tumor cells with a remarkable level of effectiveness.

### 2.3. Induction of DNA Fragmentation by IQ

One of the key events in the process of apoptosis is the fragmentation of DNA, which differentiates apoptotic cells from necrotic ones. DNA cleavage is facilitated by nucleases such as CAD (CASPASE-activated DNase), resulting in fragmented patterns visible on agarose gels (Figure 4B).

#### TUNEL Assay

In order to confirm apoptosis, flow cytometry was used to quantify double-stranded DNA fragmentation by measuring the TUNEL assay score. The DNA breakage can be detected by this classical method, mostly by labeling the nucleic acid terminal end, which was employed in the current study to determine the effects of treating HCT-116 cells with IC_50_ of IQ after 24 h of DNA fragmentation. According to the results, there was a significant increase in the percentage of DNA damage (10.4%) in the cells treated with IQ in comparison to the control cells (Figure 4C,D).

### 2.4. Reduction in ΔΨm in HCT-116 Cells Treated with IQ

**Figure 5:**
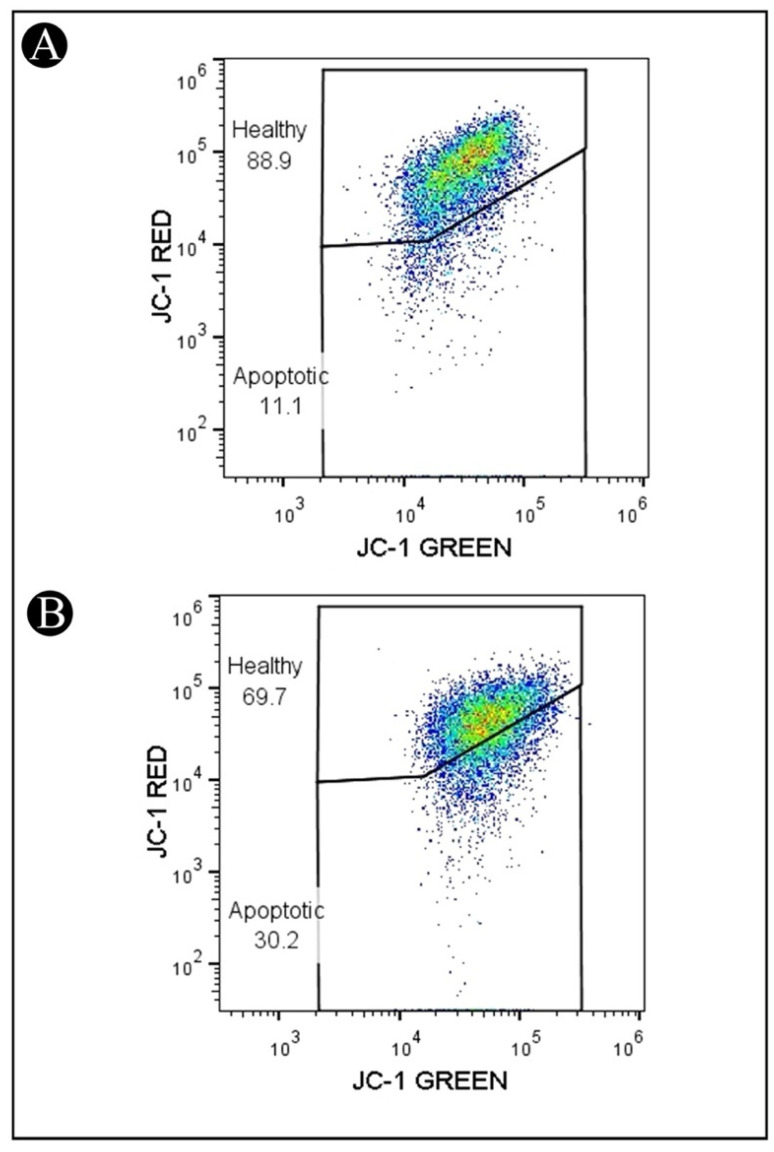
Representative flow cytometry plots of effects of IQ on mitochondrial membrane potential (ΔΨm) determined by JC-1 staining method. (**A**) Untreated. (**B**) HCT-116 cells treated with IQ (IC_50_).

A lipophilic fluorochrome known as JC-1 (5,5′,6,6′-tetrachloro-1,1′,3,3′- tetraethylbenzimidazolcarbocyanine iodide) was used to assess the mitochondrial membrane potentials (ΔΨm). The ΔΨm has been shown to be a sensitive indicator of mitochondrial permeability, so in order to determine whether IQ is involved in mitochondrial apoptosis, JC-1 staining was used to evaluate the mitochondrial membrane potential in treated cells by using a flow cytometer. As shown in Figure 5A,B, cells that were treated with IQ showed a significant decrease in JC-1 red fluorescence when compared to untreated cells. A decrease in the JC-1 red fluorescence corresponds to an increase in the depolarization of the mitochondrial membrane potential. This was indicated by a decrease in the MFI (mean fluorescence intensity) of red fluorescence and an increase in the cells in the apoptotic gate (cells with low red fluorescence). These findings indicate the mitochondrial pathway may be involved in IQ-induced apoptosis.

### 2.5. Expression of Genes Related to Apoptosis

**Figure 6:**
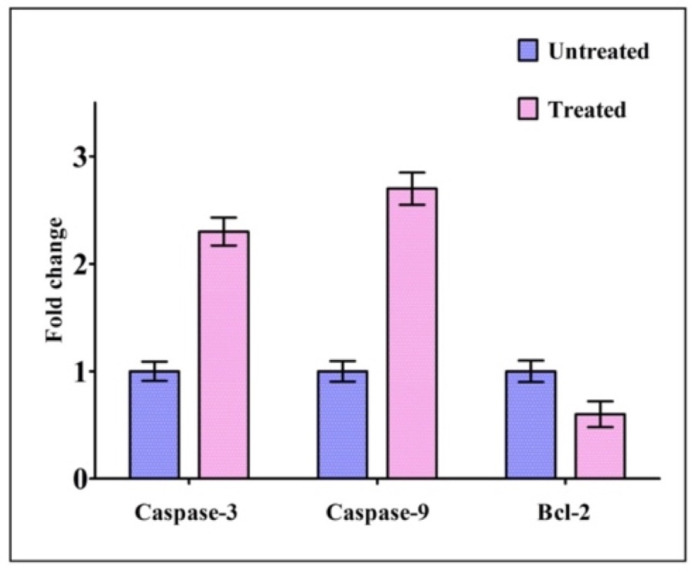
Gene expression levels in HCT-116 cells treated with the IC_50_ concentration of IQ. The expression level of apoptosis-related genes was determined via quantitative real-time PCR. GAPDH was used as an internal control. Error bars indicate the SD (standard deviation) of three independent experiments.

The expression of apoptotic genes, such as caspase-3 and caspase-9, along with Bcl-2, was detected by real-time PCR in HCT-116 cells treated with IQ in order to determine whether or not these cells are subjected to apoptosis. The expression levels of caspase-3 and caspase-9 in treated cells were found to have increased in comparison to those of untreated cells, whereas the expression levels of Bcl-2 were found to have decreased in treated cells (Figure 6).

### 2.6. Molecular Docking

**Figure 7:**
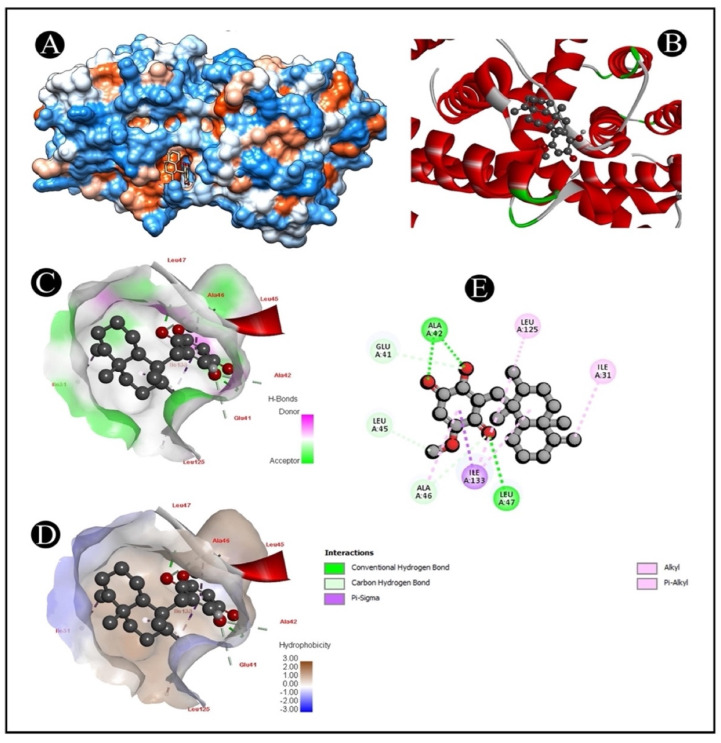
Visualization of the docking analysis of IQ binding with 4S0O. (**A**) Hydrophobicity surface 3D representation. (**B**) Interaction of IQ with 4S0O. (**C**) Visualization of hydrogen bond. (**D**) Visualization of hydrophobic interaction. (**E**) Two-dimensional representation describing the binding of IQ with an active site of 4S0O.

**Figure 8:**
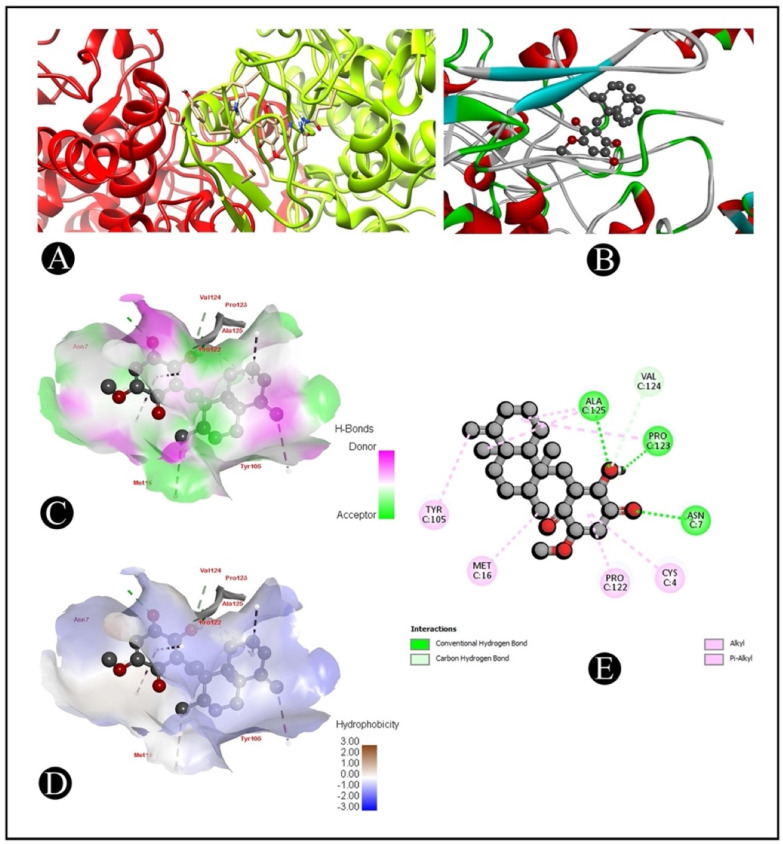
Visualization of the docking analysis of IQ binding with 1CX2. (**A**) Interaction of IQ with 1CX2. (**B**) Close-up of interaction of IQ with 1CX2. (**C**) Visualization of hydrogen bond. (**D**) Visualization of hydrophobic interaction. (**E**) Two-dimensional representation describing the binding of IQ with an active site of 1CX2.

**Figure 9:**
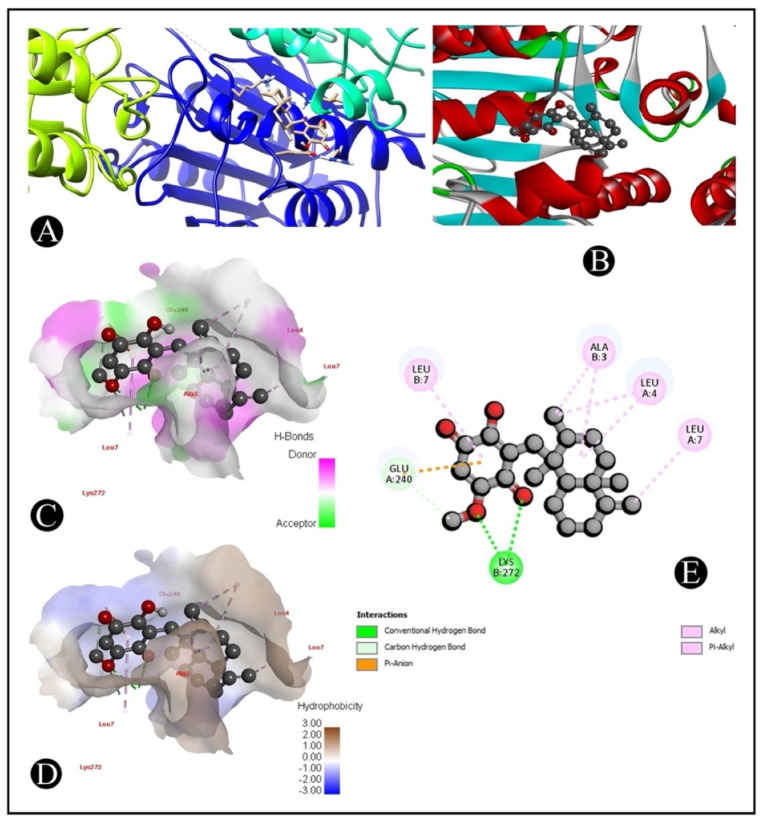
Visualization of the docking analysis of IQ binding with 2AR9. (**A**) Interaction of IQ with 2AR9. (**B**) Close-up of interaction of IQ with 2AR9. (**C**) Visualization of hydrogen bond. (**D**) Visualization of hydrophobic interaction. (**E**) Two-dimensional representation describing the binding of IQ with an active site of 2AR9.

**Figure 10:**
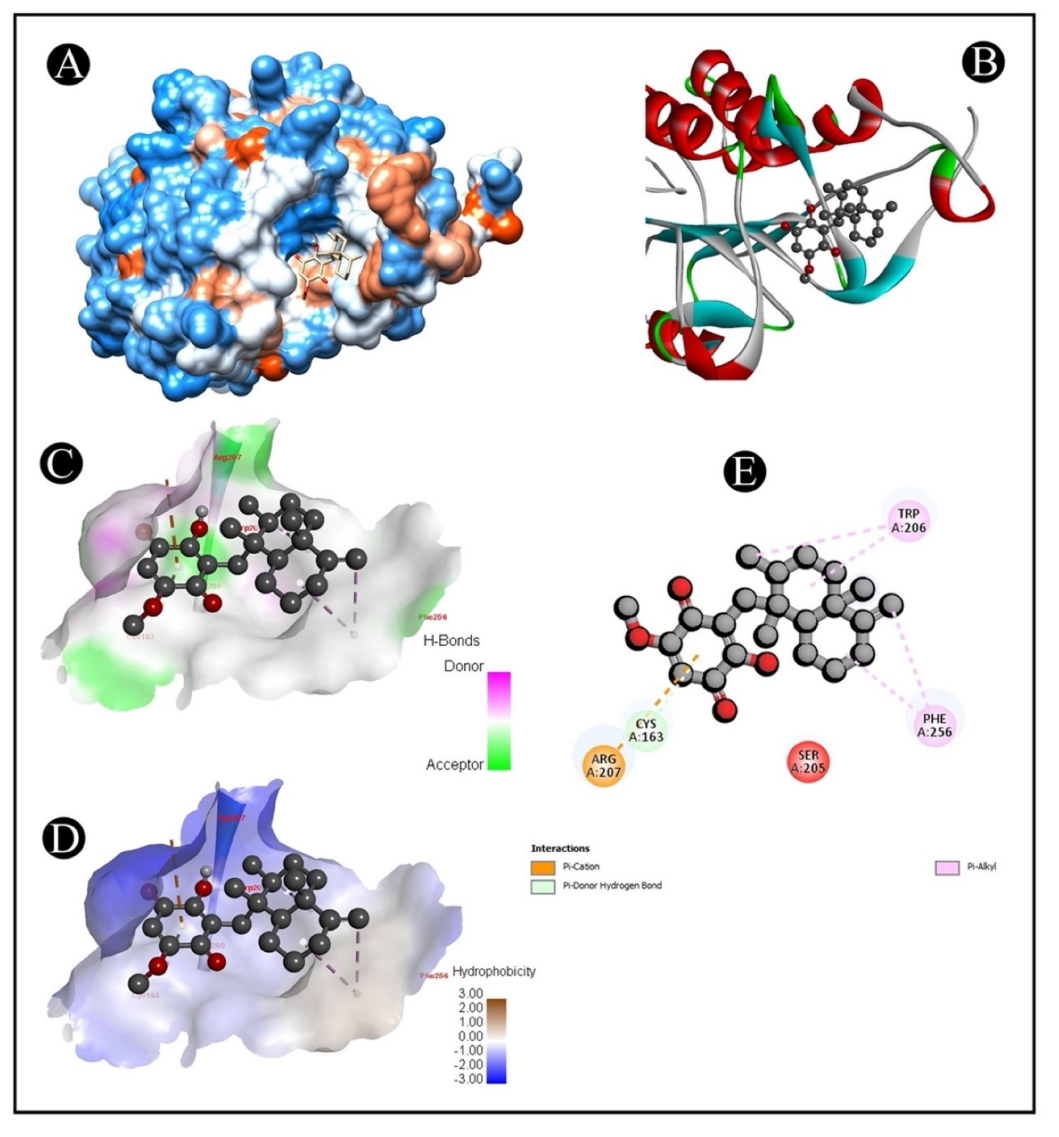
Visualization of the docking analysis of IQ binding with 5I9B. (**A**) Hydrophobicity surface 3D representation. (**B**) Interaction of IQ with 5I9B. (**C**) Visualization of hydrogen bond. (**D**) Visualization of hydrophobic interaction. (**E**) Two-dimensional representation describing the binding of IQ with an active site of 5I9B.

The AutoDock Vina was used to pair the IQ with the apoptotic signaling proteins in order to predicting receptor–protein binding modes and affinities. The low binding energy of IQ for the protein indicated that it had a high affinity for it. A molecular coupling of apoptotic signaling proteins associated with IQ shows that this protein has a high affinity for binding with proteins. According to molecular coupling studies of IQ with apoptotic signaling proteins, it binds proteins with high affinity. The top-rated pose of the ligand–receptor complex is presented in Table 1 and Table 2 along with a summary of the binding affinity of the complex. It is apparent from Figure 7, Figure 8, Figure 9 and Figure 10 that IQ occupied the active site in a variety of different ways.

## 3. Discussion

A variety of compounds are currently being investigated for their potential anticancer properties, with the aim of improving cancer treatment [3]. Nevertheless, a number of treatments have failed due to intolerance or resistance, and as a result, the disease has progressed. Over the past few years, the interest in the use of marine sponges has increased significantly because they possess a diverse range of bioactive metabolites with fewer side effects [4]. This study aimed to investigate the anticancer properties of IQ, a sesquiterpene quinine component, followed by exploring the mechanisms behind the activities of this component leading to cancerous cell death [15,16,17]. There has also been evidence that IQ possesses some anticancer activity against a few cancers, though its underlying mechanism has yet to be thoroughly elucidated. During this study, IQ was analyzed for its growth inhibition properties against the colorectal cancer cell line HCT-116. A dose-dependent inhibition of cell proliferation was observed in the results obtained with IQ.

Treatment of cancer has traditionally focused on inhibiting growth and inducing apoptosis in cancer cells, regardless of the specific sites of action of pharmacological agents [21]. As part of our investigation to determine whether the growth inhibitory effect of IQ is attributable to its apoptosis-inducing effect, AO/EtBr staining, flow cytometric analysis and DNA fragmentation analysis were conducted. Based on our observations, the morphological changes observed during cell death were consistent with apoptosis. IQ was more potent in inducing early apoptosis and late apoptosis, and fewer necrotic cells after 24 h. In early apoptotic cells, specific signals attract phagocytes without causing inflammation, whereas in late apoptotic and necrotic cells, further pro-inflammatory signals are released [22].

In one of the earliest stages of apoptosis, cells that are undergoing the process of apoptosis reorient the phosphatidylserine from the inner membrane to the leaflet on the outer membrane. In this condition, annexin V can bind to a cell surface, which can be used as a marker for the onset of apoptosis. Using the Annexin V assay, we determined whether HCT-116 cells exposed to IQ were capable of undergoing apoptosis. In the analysis, it was found that IC_50_ concentrations of IQ for 24 h resulted in early and late apoptosis cells (resulting in more late apoptotic cells than early apoptotic cells). As a result of the permeabilization of the plasma membrane, early apoptotic cells can become late apoptotic ones [23]. A very small percentage of cells died because of necrosis. This may be because the cells that died by apoptosis were later degraded because they did not have the capacity to repair their DNA during late apoptosis [24]. As a result of the apoptotic properties of IQ, cells also showed significant DNA fragmentation as well. By selectively cleaving vital substrates within the cell, caspases have been shown to induce apoptotic morphological changes as well as DNA fragmentation when activated [25,26]. Accordingly, we found that IQ causes caspase-3 activation as evidenced by the detection of caspase-3 activity in treated cells. When caspase-3 is activated, the cell initiates DNA fragmentation and apoptosis by activating enzymes such as DNase which, in turn, results in DNA fragmentation and consequently, apoptosis. As a result of our analysis, we suggest that the anticancer activity of IQ against HCT-116 cells might be mediated by the promotion of cell apoptosis.

**Figure 11:**
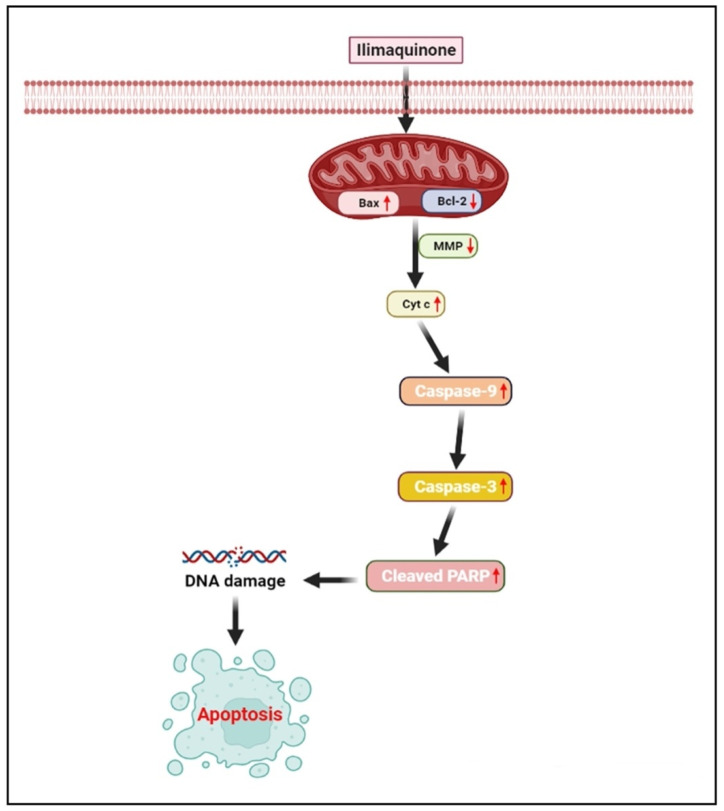
IQ induces HCT-116 cell apoptosis through the mitochondrial-related apoptosis pathway and activation of the caspase cascade.

There are two main pathways that trigger apoptosis: mitochondrial signaling (intrinsic) and death receptor signaling (extrinsic). Death receptors trigger activation of caspase-8 through Fas/FasL, which belongs to the family of tumor necrosis factor [27,28]. The mitochondrial pathway is involved in the action of several anticancer drugs using cytochrome c (cyt-c), apoptotic protease activating factor 1 and caspase-9 [29]. There are many proteins within the Bcl-2 family that regulate apoptosis in mitochondria, including pro-apoptotic proteins such as Bax, Bad, and Bak, and antiapoptotic proteins such as Bcl-2, Bcl-xL, and Bcl-w [30,31]. A combination of Bax activation and Bcl-2 inhibition leads to mitochondrial disruption, which is followed by cytochrome-c release from the outer mitochondrial membrane into the cytosol. As the apoptotic protease-activating factor 1 and cytochrome-C are associated cytosolically, they are able to activate caspase-9, which, in turn, triggers caspase-3 and caspase-7 [32,33]. One of the key executioners of apoptosis is caspase-3, which is responsible for cleaving and in activating cellular substrates such as poly (adenosine diphosphate-ribose) polymerase (PARP) [34]. The PARP is a DNA repair enzyme that has a molecular weight of b116 kDa and is triggered by DNA strand breaks. Caspase-3 activation and apoptosis have been linked to PARP cleavage [35]. In the present study, the transcriptional level of several mitochondrial apoptosis-related genes was measured. Based on our gene expression study, we identified elevated levels of caspase-3 and caspase-9, and Bcl-2 levels were significantly decreased. These results suggest that mitochondria-dependent pathways are involved in IQ-induced apoptosis in HCT-116 cells. This mechanism was further confirmed via a mitochondrial membrane potential (ΔΨm) assay. The activation of mitochondrial dysfunction as part of an early apoptotic process is characterized by alterations in the mitochondrial redox potential as well [32,33]. Based on our findings, we were able to demonstrate that IQ possesses the ability to decrease the cell’s mitochondrial membrane potential and thus lead to the mitochondrial apoptosis pathway (Figure 11). Various natural products were shown to have antitumor efficacy against different types of cancers by using mitochondrial apoptotic pathways [36,37,38,39]. 

Nowadays, approaches such as virtual screening are widely applied in drug discovery and design due to advances in technology, computational science and the integration of biological and pharmaceutical studies [40]. A structure-based virtual screening method is a virtual screening technique that makes use of molecular docking in order to model and analyze the binding relations between a ligand and a target molecule in order to optimize the drug discovery process [41]. Molecular docking is one of the latest tools for designing stable and effective interactions between ligands and receptors, as well as predicting the optimal combination of ligands and receptors [42]. There are two fundamental aspects of docking programs, which are search algorithms and the scoring function, both of which are fundamental to a successful docking. A search algorithm can be regarded as a process that can lead to a more complete understanding of the best methods for docking a ligand onto a molecular target amongst the myriad configurations of ligands. In view of the fact that a vast number of binding modes are found between a ligand and a biological target molecule, a search algorithm cannot only be used to consider the optimal orientation of the ligand with reference to the target, but can also be used to make docking as economical and time-efficient as possible, as well as to provide the optimal orientation of the ligand with respect to the target [43]. Moreover, in the development of novel drugs, the use of molecular docking programs has become an increasingly popular method of in silico drug screening, partly because it is quicker and less expensive than traditional laboratory experiments, and also because it is a faster and cheaper method of refining drugs than physical screening. A computational protein–ligand docking technique was utilized in this study in order for the candidate ligand IQ to interact with several apoptotic signaling target proteins (Table 2). As a whole, we demonstrated the binding energy between IQ and apoptotic proteins, hydrogen bond interactions and hydrophobic interactions for further analysis.

## 4. Materials and Methods

### 4.1. Cell Culture

Human colorectal cancer cell line HCT-116 was gathered from the National Centre for Cell Science (NCCS), situated in Pune, India. The DMEM medium supplemented with 10% fetal bovine serum (Hi-Media^®^, Mumbai, India), 100 units of penicillin (U/mL) and 100 (µg/mL) of streptomycin was used for culturing the cells. The cells were maintained at 37 °C in a humidified atmosphere of 5% CO_2_.

### 4.2. Cell Viability Assay

The MTT assay was used to determine the effects of IQ on viability of HCT-116 cancer cells. In order to harvest cells from T-25 flasks, cells were trypsinized and aspirated into a 5 mL centrifuge tube. Cells were collected via centrifuging at 300× *g*. By using culture medium, the cell count was adjusted so that approximately 10,000 cells were in 200 μL of suspension. Each well of the 96 well microtiter plate was filled with 200 μL of the cell suspension, and the plate was incubated at 37 °C in 5% CO_2_ for 24 h. Aspiration of the spent medium was carried out after 24 h. In order to treat the cells, 200 μL of IQ at different concentrations (1.25 µM, 2.5 µM, 5 µM, 10 µM and 20 µM) was introduced into the wells, and the plates were further incubated at 37 °C in 5% CO_2_ atmosphere for 24 h. Each well was then filled with 200 μL of medium containing 10% MTT reagent, and then incubated for 3 h at 37 °C under 5% CO_2_. The culture medium was removed completely without disturbing the crystals that had formed in the medium. Finally, 100 μL of solubilization solution (DMSO) was added to the plate and agitated gently in a gyratory shaker to dissolve the formazan. A microplate reader was used to measure the absorbance at 570 nm and 630 nm. Using the dose–response curve for the cell line, we calculated the concentration of test drug required to inhibit the cell growth by 50% (IC_50_) after subtracting the background and blank [44].

### 4.3. Acridine Orange–Ethidium Bromide (AO/EB) Staining

In 6-well plates, cells were plated at a density of 3 × 10^5^ cells/per 2 mL, followed by incubation in a CO_2_ incubator at 37 °C for 24 h. Cells were then washed with 1 mL of 1X PBS after aspiration of spent medium. Following treatment with IQ (IC_50_), the cells were further incubated for 24 h. After incubation, the medium was then removed and washed in cold PBS. As the next step, 500 µL of AO/EB staining solution (10 µL of AO (2 mg/mL) and 10 µL of EB (2 mg/mL) in 1 mL of PBS) was added to the cells. The mixture was thoroughly mixed and incubated for 5 min. A fluorescent microscope (XDFL series, Sunny Instruments, China) was used to take images immediately after washing the cells in PBS three times [45].

### 4.4. Annexin V–PI Apoptosis Assay

Cell pre-treatment in 6-well plates was carried out as described above. After removal of the spent medium, 1 mL of PBS was added. Then, IQ (IC_50_) was added to 2 mL of culture medium and cells were treated for 12–16 h. As a negative control, wells were left untreated. After incubation, medium was collected from each well, transferred to 5 mL centrifuge tubes and washed with 500 µL of PBS. Immediately, after removing the PBS solution from the sample, 200 µL of trypsin-EDTA solution was added and incubated at 37 °C for 3–4 min. After a few minutes, the wells were filled with medium again, and the cells were harvested into centrifuge tubes. The centrifugation was performed at 300× *g* for 5 min at 25 °C. Following centrifugation, the supernatant was removed and washed twice with PBS. After removing the PBS, cells were resuspended in 1X binding buffer at a concentration of 1 × 10^6^ cells/mL. In the following step, 100 µL of cell suspension was transferred into a 5 mL tube, and Annexin V–AbFlour 488 was added. For optimal results, cells were gently mixed and incubated for 15 min in the dark at 25 °C. In the end, 400 µL of 1X binding buffer and 2 µL of PI were added and mixed thoroughly. Cells were analyzed by flow cytometry immediately after PI was added [46].

### 4.5. DNA Fragmentation Assay

The HCT-116 cells (3 × 10^5^ cells/well) were cultured in 6-well plates, and then treated for 24 h with IQ (IC_50_). Using standard phenol–chloroform extraction procedures, DNA was extracted. Tris-EDTA (TE) buffer was used to dissolve the extracted DNA, and then 2% agarose gels were used to analyze the DNA. To visualize DNA, the gel was stained with ethidium bromide and photographed using a gel doc system [47].

### 4.6. Apoptotic DNA Degradation Assay by TUNEL (Terminal Deoxynucleotidyl Transferase dUTP Nick End Labeling) Assay

In accordance with the manufacturer’s instructions, DNA degradation during apoptosis was detected by the TUNEL assay using APO-DIRECTTM Kit (Pharmingen, BD Biosciences, San Diego, CA, USA) provided by the manufacturer. Cell pre-treatment in 6-well plates was carried out as described above. Cells were washed with 1 mL of 1X PBS after aspiration of spent medium. In the dark, cells were fixed in 4% paraformaldehyde solution for 1 h after being treated with IQ (IC_50_) for 24 h. For permeabilization, the cells were washed with PBS and incubated on ice for 20 min in a solution containing 1% Triton X-100 and 0.1% sodium citrate. In humidified air at 37 °C and in the dark, DNA was then labeled by incubating the cells with TUNEL reaction mixture (Tdt enzyme and fluorescein-conjugated dUTP) for 60 min. The percentage of TUNEL-positive cells was determined using flow cytometry by analyzing the histograms generated [48].

### 4.7. Mitochondrial Membrane Assay

Cell pre-treatment in 6-well plates was carried as described above. Following the aspiration of the spent medium, 1 mL of PBS was added. Then, the cells were treated with IQ (IC_50_) and further incubated for 24 h. Following incubation, the medium was withdrawn from all the wells and transferred into centrifuge tubes and washed 500 µL of PBS. Following the removal of PBS, 200 µL of trypsin-EDTA solution was added and incubated for 3–4 min at 37 °C. In the following step, cells were harvested directly into centrifuge tubes by pouring the culture medium back into the respective wells. A centrifuge was performed at 300× *g* at 25 °C for 5 min, and the supernatant was discarded. After washing the pellet twice with PBS, 0.5 mL of freshly prepared JC-1 working solution was added and incubated in a CO_2_ incubator at 37 °C for 10–15 min. After incubation, cells were washed twice with 1X assay buffer and then centrifuged for 5 min at 400× *g*. After centrifugation, cells were resuspended in 0.5 mL of 1X assay buffer and analyzed by flow cytometry [49].

### 4.8. Analysis of the Expression of Genes Involved in Apoptosis

According to the manufacturer’s instructions, cellular RNA was isolated using Tri-Pure Isolation Reagent (Sigma-Aldrich^®^, Bangalore, India). To quantify the amount of RNA obtained, 1.2% agarose gels were electrophoresed, stained with ethidium bromide and photographed under ultraviolet light. RNA was reverse-transcribed using the RT-first strand synthesis kit from Qiagen (Valencia, CA, USA). To measure gene expression levels relative to controls, SYBR green qRT PCR was used (Applied Biosystems^®^ 7500 Fast Real-Time PCR machine, Foster City, CA, USA). Data were analyzed using the ΔΔCt method, with fold changes expressed over control groups. As shown in Table 3, the primer pairs were used separately. In order to determine relative gene expression, the following conditions were used: reverse transcription for 45 min, one cycle of 95 °C and 10 min hold, followed by 40 cycles of 95 °C and 15 s hold. The annexed temperature (caspase-3 and caspase-9 and Bcl-2 and GAPDH) was 60 °C, and a 60 s hold was used [50].

### 4.9. Molecular Docking Analysis

Crystal structures of several apoptotic signaling proteins, such as BAX (PDB: 4S0O.pdb), caspase-3 (PDB: 5I9B.pdb), COX-2 (PDB: 1CX2.pdb) and caspase-9 (PDB: 2AR9.pdb), known to be associated with CRC were retrieved from Protein Data Bank (RCSB PDB). An eminent database, PubChem, was used for the retrieval of the three-dimensional structure of IQ, and Open Babel was used to convert this structure to the PDB format [51]. In order to dock the IQ with the receptor structure individually (BAX, caspase-3, COX-2 and caspase-9), molecule docking software AutoDock 4.2.6 was used [52]. This study used a similar docking protocol to that implemented in previous studies [53,54]. Using an auto grid [52], the grid map using a grid box was prepared. The grid size was set to 125 × 106 × 156 xyz points for 1CX2, 97.81 × 81.33 × 125.13 xyz points for 2AR9, 75.64 × 41.01 × 50.63 xyz points for 4S0O receptors and 54.76 × 55.66 × 50.62 xyz points for 5I9B. The grid center for 1CX2 was designated at dimensions (x, y and z) 42.780, 34.762 and 37.574; for 2AR9 at (x, y and z) 21.40, 35.16 and 22.79; for 4S0O at (x, y and z): 20.58, −0.17 and 21.40; and for 5I9B at (x, y and z): −6.70, −19.22 and −10.84. The grid box was designed to enclose both receptor binding sites and provide adequate space for ligand translation and rotation. Predicted binding energies of docked conformations were ranked using UCSF Chimera [55] in order to determine the intermolecular hydrogen bonding of active-site amino acid residues [56].

## 5. Conclusions

Based on the results of the present study, it can be concluded for the first time that IQ triggers the process of apoptosis (programmed cell death) in HCT-116 carcinoma cells. There appears to be a requirement of a 24 h treatment period in order to achieve the effect of inducing apoptosis. As it turns out, IQ is more effective at inducing apoptosis, since it delivers a higher degree of efficacy at lower concentrations as compared to the other apoptosis-inducing agents. According to the research, mitochondrial pathways and caspase-3-mediated pathways are involved in IQ’s pro-apoptotic effects, which may explain the pro-apoptotic effects of IQ. As a whole, the present study offers valuable mechanistic insights into the mechanisms through which IQ exerts its anticancer effects on tumor cells. These preliminary data are encouraging and may contribute to the development of new chemotherapeutic agents based on the marine environment in the near future that could be used to treat colorectal cancer and other undesirable and life-threatening cancers that are difficult to treat.

## Figures and Tables

**Table 1:** Binding affinities of top-rated pose of ligand–receptor complex.

Compound Name	5I9B	1CX2	2AR9	4S0O
Ilimaquinone	−6.9	−8.9	−8.1	−8.2

**Table 2:** Binding affinities of top-rated pose of ligand–receptor complex.

Receptor–Ligand	Receptor–Ligand Interactions	Distance in Angstroms
**2AR9–Ilimaquinone**	(B:LYS272) HZ1-O (Ligand)	2.50
	(B:LYS272) HZ3-O (Ligand)	2.53
	(B:LYS272) HZ3-O (Ligand)	2.75
	(B:LYS272) CE-O (Ligand)	3.51
	(A:GLU240) OE1-C (Ligand)	3.64
	(A:GLU240) OE1-Pi Anion interaction	3.41
	(A:LEU4) Alkyl–Alkyl interaction	4.49
	(B:ALA3) Alkyl–Alkyl interaction	4.74
	(B:ALA3) Alky–C Alkyl interaction	3.94
	(A:LEU4) Alkyl–C Alkyl interaction	4.23
	(A:LEU7) Alkyl–C Alkyl interaction	3.99
	(B:LEU7) Pi-Alkyl interection	4.77
**1CX2–Ilimaquinone**	(ASN7) HD21-O (Ligand)	2.56
	(ALA125) HN-O (Ligand)	2.35
	(PRO123) O-H (Ligand)	1.67
	(VAL124) CA-O (Ligand)	3.57
	(PRO123) Alkyl–Alkyl interaction	5.22
	(ALA125) Alkyl–Alkyl interaction	4.27
	(ALA125) Alkyl–C Alkyl interaction	3.86
	(MET16) Alkyl–C Alkyl interaction	4.69
	(TYR105) Pi-Alkyl interaction	4.06
	(CYS4) Pi-Alkyl interaction	5.02
	(PRO122) Pi-Alkyl interaction	4.67
**4S0O–Ilimaquinone**	(ALA42) HN-O (Ligand)	2.44
	(ALA42) HN-O	2.25
	(LEU47) HN-O	2.27
	(GLU41) CA-O	3.61
	(ALA42) CA-O	3.57
	(ALA46) CA-O	3.54
	(LEU45) O-C	3.29
	(ILE133) CD1 Pi Sigma interaction	3.50
	(ILE133) Alkyl–Alkyl interaction	4.88
	(LEU125) Alkyl–C Alkyl interaction	4.78
	(ILE33) Alkyl–C Alkyl interaction	5.02
	(ILE31) Alkyl–C Alkyl interaction	3.90
	(ALA46) Pi-Alkyl interaction	4.47
**5I9B–Ilimaquinone**	(ARG207) NH1-Pi interaction	4.41
	(CYS163) SG-Pi interaction	3.70
	(TRP206) Pi-Alkyl interaction	5.06
	(TRP206) Pi-C Alkyl interaction	4.65
	(PHE256) Pi-Alkyl interaction	4.11
	(PHE256) Pi-C Alkyl interaction	3.87

**Table 3:** Sequences of primers of apoptosis regulatory genes.

Gene	Forward Primer	Reverse Primer
GAPDH	5′ CATGGGGAAGGTGAAGGTCGA 3′	5′ TTGGCTCCCCCCTGCAAATGAG 3′
Bcl-2	5′ TTCGATCAGGAAGGCTAGAGTT 3′	5′ TCGGTCTCCTAAAAGCAGGC 3′
Caspase-3	5′ TGCGCTGCTCTGCCTTCT 3′	5′ CCATGGGTAGCAGCTCCTTC 3′
Caspase-9	5′ CCAGAGATTCGCAAACCAGAGG 3′	5′ GAGCACCGACATCACCAAATCC 3′

## Data Availability

All data are available in the manuscript.
